# Benzodiazepine‐receptor agonist prescription in a population of hospitalised patients in four psychogeriatric units in Switzerland

**DOI:** 10.1111/jsr.14317

**Published:** 2024-08-07

**Authors:** Maria Dalmau i Ribas, Julien Sauser, Estelle Gillès de Pélichy, Montserrat Méndez Rubio, Jean‐Pierre Schuster, Armin Von Gunten, José Haba‐Rubio

**Affiliations:** ^1^ Service Universitaire de Psychiatrie de l'Age Avancé, Département de Psychiatrie Centre Hospitalier Universitaire Vaudois Lausanne Switzerland; ^2^ Centre d'Investigation et de Recherche sur le Sommeil Centre Hospitalier Universitaire Vaudois Lausanne Switzerland; ^3^ Center of Clinical Research (CRC), Training and Research Department Centre Hospitalier Universitaire Vaudois Lausanne Switzerland; ^4^ Centre de Psychiatrie et Psychothérapie Lémanique Morges Switzerland

**Keywords:** benzodiazepine, elderly, hospital, inpatient, insomnia symptoms, psychiatry, psychogeriatrics, sedative‐hypnotic, z‐drug

## Abstract

The aim of this study is to describe the patterns of prescription of benzodiazepine‐receptor agonists in hospitalised patients in four psychogeriatric units in Switzerland. This is a retrospective cross‐sectional study that included patients aged 65 years or more hospitalised in one of the four psychogeriatric units of a university hospital in Switzerland during 2019. The presence, type and dose of benzodiazepine‐receptor agonists was assessed at admission and at discharge. Three‐hundred and eighty‐six patients (214 women, 78.2 ± 8.1 years) were included in the study; 33.4% of patients had at least one benzodiazepine‐receptor agonist at admission and 22.5% at discharge. The relative reduction of benzodiazepine‐receptor agonists prescription in standardised dose was 78%. Age was found to be a protective factor against benzodiazepine‐receptor agonists prescription at admission (adjusted odds ratio 0.94, confidence interval 0.91–0.98), and diagnosis of substance abuse was found to be a risk factor (adjusted odds ratio 4.43, confidence interval 1.42–17.02). Longer hospital stays (> 14 days) were associated with higher reduction of benzodiazepine‐receptor agonists. The prevalence of a prescription of benzodiazepine‐receptor agonists at admission was high, but during the psychogeriatric hospitalisation benzodiazepine‐receptor agonists prescription decreased both in absolute and relative terms.

## 1 INTRODUCTION

Benzodiazepines and z‐drugs, also known as benzodiazepine‐receptor agonists (BZRA), are widely prescribed among older people as a treatment for insomnia symptoms (Bell et al., 2007; Markota et al., 2016), despite recommendations advising against their use for this indication (Luta et al., 2020; Pottie, 2018), especially among older adults (Gerlach et al., 2018). Older people have a higher risk of adverse effects from BZRA, which include increased risk of falls, fractures, addiction, cognitive impairment and overall mortality (Hill & Wee, 2012; Palmaro et al., 2015). Moreover, patients suffering from dementia are at highest risk of adverse effects (Rochon et al., 2017). Furthermore, the long‐term efficacy of these treatments is limited, with almost 60% of their effect being explained by the placebo effect (Winkler & Rief, 2015). For all these reasons, inappropriate prescription of BZRA in the general population, but more specifically among the elderly, can be considered a public health threat (Soong et al., 2019). The first‐line treatment for insomnia disorder according to current guidelines is cognitive behavioural therapy for insomnia (CBT‐I; Riemann et al., 2023), although specific guidelines on how to manage acute insomnia symptoms do not exist, and current guidelines recommending CBT‐I are generally not applied in psychiatric facilities (Schneider et al., 2023).

Despite the available strategies for deprescribing BZRA in the elderly (Markota et al., 2016; Pottie, 2018; Soong et al., 2019), several barriers to modifying the current prescribing models still exist (Anderson et al., 2014). Most of the studies describing this problem have focused on the general population, but few studies focus on prescribing patterns of BZRA among hospitalised patients, although new prescriptions during and after the hospitalisation are very frequent (Bell et al., 2007). Insomnia symptoms are very common in the general population, with about 34% of the European adults presenting with insomnia symptoms (Hafner et al., 2023) and about 10% meeting the diagnostic criteria for insomnia disorder (Riemann et al., 2023). The prevalence of insomnia symptoms is even higher among hospitalised patients because of environmental (noise, light, discomfort) and patient‐related factors (anxiety, pain; Fan‐Lun et al., 2019; Heinemann et al., 2019). For this reason, sleep‐inducing drugs are widely prescribed among inpatients (Pillai et al., 2017), with about 40% of Swiss inpatients receiving at least one sleep‐inducing drug during their hospitalisation (Schumacher et al., 2017). The use of these drugs among patients with psychiatric comorbidities is much higher than that of the general population (Abolhassani et al., 2019), as about 90% of psychiatric inpatients report insomnia symptoms during hospitalisations (Hartwig et al., 2019).

To the best of our knowledge, there are no studies describing the patterns of BZRA prescription among older patients hospitalised in psychiatry, in spite of the fact that because of their age and their psychiatric comorbidities they are more likely to receive these drugs and to suffer from adverse effects (Abolhassani et al., 2019; Palmaro et al., 2015; Rochon et al., 2017). The present study describes the patterns of prescription of BZRA among hospitalised patients in four psychogeriatric units from a university hospital in Switzerland.

## 2 METHODS

### 2.1 Aim

The primary aim of this study is to describe the patterns of BZRA prescription among patients hospitalised in four psychogeriatric units in Switzerland (Centre Hospitalier Universitaire Vaudois), and to study the changes that occurred during the hospitalisation in absolute terms (percentage of prescription at admission and at discharge), in relative terms (standardised dose of BZRA at admission and at discharge), and in terms of the choice of drug (changes between admission and discharge).

Secondary aims include studying associations between BZRA prescription at admission and patients' characteristics to identify possible risk and protective factors, studying changes in BZRA prescription depending on length of stay (LOS), and studying associations between BZRA reduction and patients' characteristics to identify possible factors predicting change.

### 2.2 Design

This is a retrospective cross‐sectional study that included all patients aged 65 years or more having been hospitalised in one of the four psychogeriatric units of a Swiss university hospital for at least 3 nights between 1 January 2019 and 31 December 2019. Although the study took place in 2022, the studied year was 2019 to avoid any possible interference with the SARS‐CoV‐2 pandemics.

For the purpose of this study, we considered that the treatment at admission was the treatment on the second day of the hospitalisation, and the treatment at discharge was the treatment on the penultimate day to avoid incomplete treatments on first and last day due to late patient's arrival or early departure, respectively.

### 2.3 Participants

For ethical reasons, only patients who agreed on the use of their data for research purposes and patients who were not opposed to it were included in the study. If a patient had been admitted multiple times during the study period, only the first hospitalisation was taken into account.

### 2.4 Data collection

Data were extracted from the electronic medical information system by the hospital's data management services. Extracted data included gender, age, main diagnosis, diagnosis of dementia or mild cognitive impairment (MCI), BZRA status at admission (second day) and at discharge (penultimate day) in terms of class (benzodiazepine or z‐drug), type of molecule and dose, LOS, and unit of hospitalisation.

### 2.5 Definitions

Molecules were classified into two categories: benzodiazepines (BZD) and z‐drug. A third category, BZRA, consisted of BZD and z‐drug. The amount of prescription at admission (2nd day) and discharge (penultimate day) were defined in two different units: the number of different molecules prescribed; and the standardised dose (standardised with respect to diazepam). The standardised dose was calculated using an equivalence table (Table 1) that was designed for the purpose of this study, based on scientific literature on the subject (Altamura et al., 2013; Grandjean et al., 2021; Shader & Greenblatt, 1997; Strand et al., 2017). The total dose administered to a patient (as opposed to prescribed) at admission and discharge was accounted for.

**TABLE 1:** Equivalence table for BZDs and z‐drugs.

Diazepam	5 mg
Midazolam	3.75 mg
Triazolam	0.25 mg
Oxazepam	15 mg
Temazepam	10 mg
Lormetazepam	1 mg
Lorazepam	1 mg
Alprazolam	0.5 mg
Bromazepam	3 mg
Nitrazepam	5 mg
Clonazepam	0.25 mg
Prazepam	20 mg
Flurazepam	15 mg
Clorazepate	7.5 mg
Chlordiazepoxide	10 mg
Estazolam	1 mg
Temazepam	10 mg
Flunitrazepam	0.5 mg
Ketazolam	7.5 mg
Zolpidem	5 mg
Zopiclone	7.5 mg
Zaleplon	10 mg
Eszopiclone	1.5 mg

All values are equivalent to 5 mg of diazepam. Adapted from Grandjean et al. (2021), Strand et al. (2017), Altamura et al. (2013) and Shader & Greenblatt, 1997.

Patients' primary diagnosis was classified into 10 categories according to the International Classification of Diseases (ICD‐10; World Health Organisation, 1993): F0: organic, including symptomatic, mental disorders; F1: mental and behavioural disorders due to psychoactive substance use; F2: schizophrenia, schizotypal and delusional disorders; F3: mood (affective) disorders; F4: neurotic, stress‐related and somatoform disorders; F5: behavioural syndromes associated with physiological disturbances and physical factors; F6: disorders of adult personality and behaviour; F7: mental retardation; F8: disorders of psychological development; F9: behavioural and emotional disorders with onset usually occurring in childhood and adolescence.

The presence of dementia was considered if the diagnoses F00.x, F01.x, F02.x, F03.x, F04.x and F05.x, except F05.8 and F05.9, were present either as a primary or secondary diagnosis. The presence of cognitive impairment was considered if the diagnosis F06.7 was present either as a primary or secondary diagnosis. Patients without any of these diagnoses were considered to have no cognitive impairment.

The university hospital of the study consisted of four different psychogeriatric units: two general psychogeriatric units (GEN1 and GEN2, respectively), one unit specialised in psychiatric conditions of the elderly (PSY), and one unit specialised in dementology (DEM).

### 2.6 Ethical considerations

Ethical clearance was obtained by the Commission Cantonale d'Éthique de la Recherche sur l'Être Humain (CER‐VD), the regional ethics committee responsible for authorising human research in our area (reference number: CER‐VD 2021–00150). The study was performed in agreement with the Declaration of Helsinki, and in accordance with the applicable Swiss legislation.

### 2.7 Data analysis

Statistical analysis was performed using R version 4.2.1. Descriptive results were expressed as frequency and percentage for categorical variables, and as mean ± standard deviation (SD) for continuous variables.

Prescriptions of BZRA at admission and discharge were first compared using McNemar tests considering a dichotomisation (no prescription versus at least one prescription). Paired *t*‐tests were then performed for patients experiencing a change in standardised dose only on a log‐scale to handle right‐skewed data. The estimates are thus ratios of geometric means.

Associations between BZRA prescription at admission and patients' characteristics were analysed using a logistic regression adjusting for sex, age, primary diagnosis, secondary diagnosis of dementia or MCI, and unit of hospitalisation. Results are presented as adjusted odds ratio (aOR), 95% confidence interval (CI) and *p*‐value. Only *p*‐values < 0.05 were considered as statistically significant. When < 10 patients belonged to a diagnostic category, the category was removed because of separability.

To study the association between hospital stay characteristics and the change of prescription measured in standardised dose, various considerations needed to be accounted for. Indeed patients may either have a prescription of BZRA or not. Patients without prescription at admission could only have an increase of prescription (either null or positive). However, patients with prescription of BZRA at admission could experience either a decrease, an increase or no change. Hence, two analyses were conducted for patients receiving BZRA at admission and those without prescription. For patients without prescription of BZRA at admission, a logistic regression considering the change as a binary variable with value “no change” and “positive change” was conducted. The analysis adjusted only for the hospital LOS and the unit of hospitalisation, as the dose of BZRA was null for those patients. The linear effect of LOS was assessed using a likelihood ratio test comparing with a model using natural cubic splines with 4 degrees of freedom. The reason for not using a linear regression was the large amount of zeroes (no change). For patients with prescription of BZRA at admission, a linear regression was conducted adjusting for hospital LOS, unit of hospitalisation, and the standardised dose of BZRA at admission. Linearity was confirmed for the initial dose of BZRA, but not for the LOS. To address the non‐linearity, descriptive statistics suggested a larger reduction in change for patients staying more than 14 days, which also seemed clinically relevant. Hence, LOS was dichotomised into LOS less or equal to 14 days and more. In addition, two extreme values of change were identified and exceeded 3 standard deviations, and were thus removed.

Associations between BZRA reduction and hospital stay characteristics were analysed using a multiple linear regression adjusting for BZRA at admission, the LOS (≤  4 days versus > 14 days), and the unit of hospitalisation. Results are presented as the difference in standardised dose between admission and discharge, 95% CI and *p*‐value.

## 3 RESULTS

### 3.1 Patients' baseline characteristics

From 431 eligible patients, 386 patients were included in the study (55.4% female, mean age 78.2 ± 8.1 years). The primary diagnosis was F0 for 163 patients (42.2%), F3 for 111 patients (28.8%), F2 for 40 patients (10.4%), F4 for 31 patients (8%), F6 for 21 patients (5.4%), F1 for 18 patients (4.7%), F7 for 1 patient (0.3%) and F8 for 1 patient (0.3%). No patients had a primary diagnosis of F5 or F9. The diagnosis of dementia was retained in 210 patients (54.4%), and MCI in 31 patients (8%). The mean LOS was 39 ± 29.2 days.

### 3.2 BZRA prescription at admission and at discharge

Overall, 129 (33.4%) patients received at least one BZRA at admission. One‐hundred and twelve (29%) patients received at least one type of BZD and 33 (8.5%) received at least one type of z‐drug. Of the 129 patients receiving at least one BZRA at admission, 108 (83.7%) received only one type of molecule, 18 (14%) received two, and three (2.3%) received three.

Overall, 87 (22.5%) patients received at least one BZRA at discharge. Seventy‐two (18.7%) patients received at least one type of BZD and 28 (7.3%) received at least one type of z‐drug. Of the 90 patients receiving at least one BZRA at discharge, 70 (80.5%) received only one type of molecule, 16 (18.4%) received two, and one (1.1%) received three.

### 3.3 Changes in BZRA prescription between admission and discharge

Changes in BZRA prescription between admission and discharge were analysed: (1) in absolute terms (number and % of patients receiving BZRA at admission versus at discharge); (2) in relative terms (changes in standardised dose between admission and discharge); and (3) types of molecule at admission versus at discharge.

In absolute terms, the proportion of patients who stopped BZRA intake during the hospital stay (66/132 = 50%) was statistically significantly higher than the proportion of patients who had BZRA introduced during the hospital stay (24/261 = 9.2%; McNemar test *p* < 0.001).

**FIGURE 1:**
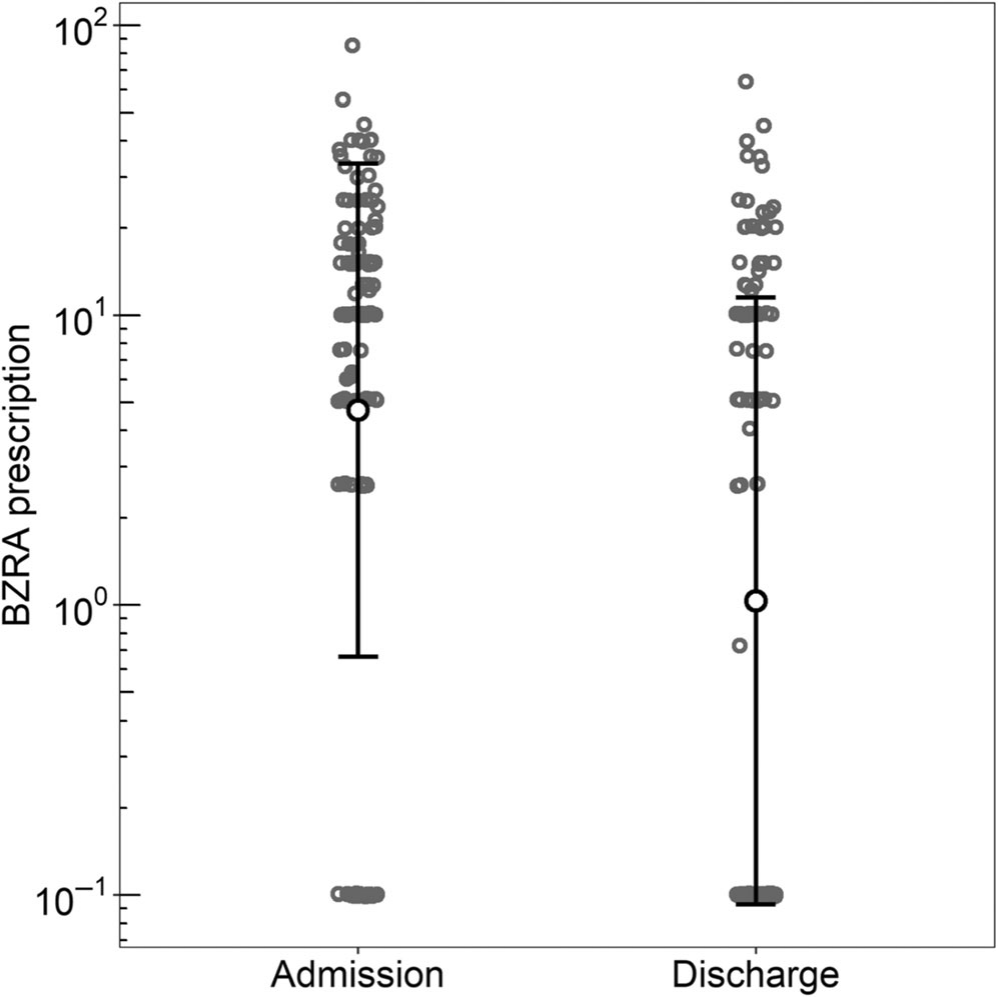
Benzodiazepine‐receptor agonists (BZRA) prescription in standardised dose (mg of diazepam) at admission and at discharge. Geometric mean and standard deviation (SD) are represented.

In relative terms, the mean standardised dose for patients with non‐zero change was 12.39 ± 13.21 mg of diazepam at admission and 7.11 ± 10.59 mg of diazepam at discharge. The ratio of geometric means was 0.22 (CI 0.12–0.41, *p* < 0.0001), indicating a statistically significant reduction of prescription of BZRA at discharge compared with admission, with a relative reduction of 78% (1–0.22 = 0.78), as shown in Figure 1.

**FIGURE 2:**
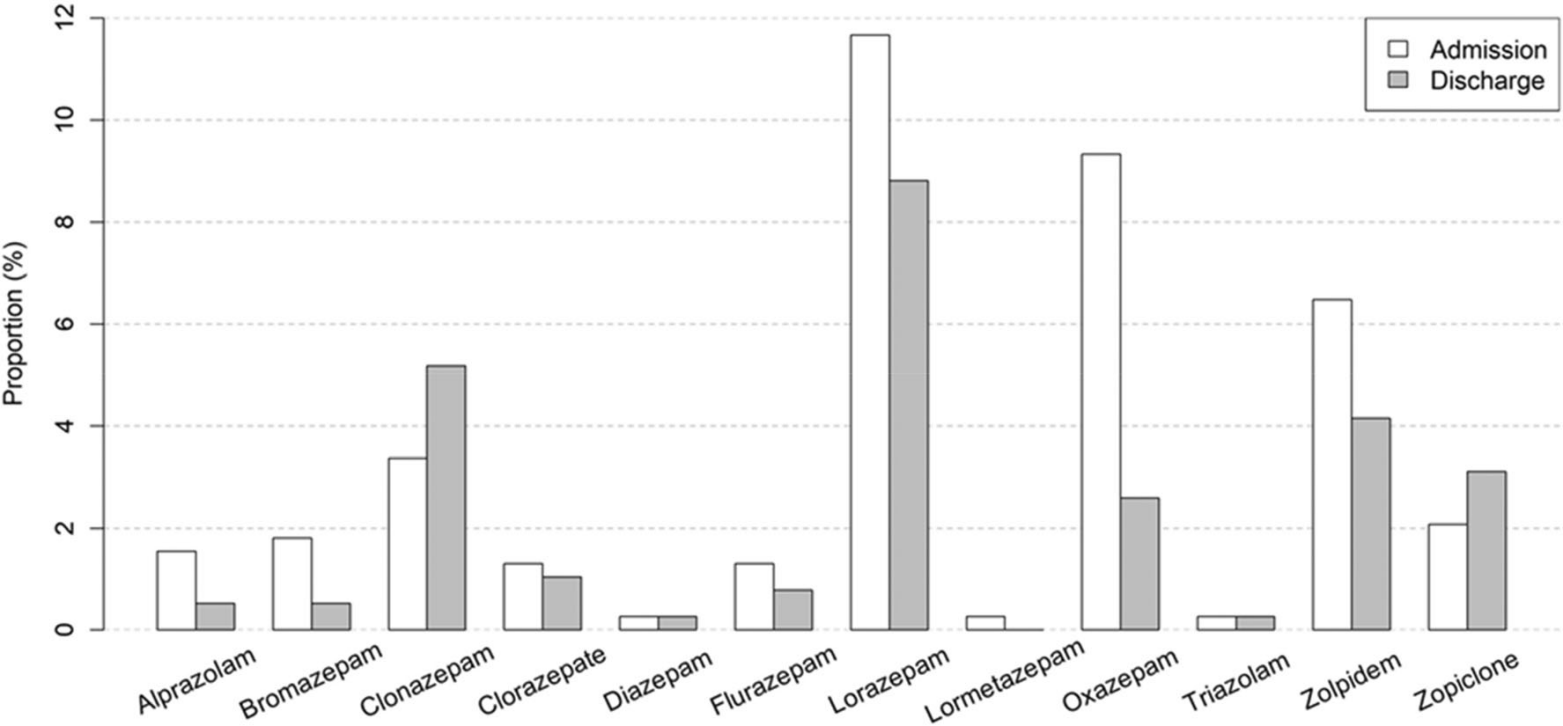
Proportion of patients (%) with a specific benzodiazepine‐receptor agonists (BZRA) molecule at admission and at discharge.

Concerning the types of molecules, Figure 2 illustrates the changes in molecules between admission and discharge. At admission, the most frequently prescribed molecules were Lorazepam (11.5% of all patients), Oxazepam (9.7%), Zolpidem (6.4%), Clonazepam (3.3%) and Zopiclone (2%). At discharge, the most frequently used molecules were Lorazepam (8.7%), Clonazepam (5.1%), Zolpidem (4.1%), Zopiclone (3.1%) and Oxazepam (3.1%). Although the most frequently used molecules were the same ones, a diminution in the total number of prescriptions was observed for all molecules, except for Clonazepam, which experienced an increase. Oxazepam was the molecule that experienced the most pronounced reduction.

### 3.4 Determinant factors of BZRA prescription at admission

Baseline characteristics and determinant factors for BZRA prescription at admission are presented in Tables 2 and 3, respectively. Diagnosis of F1 was found to be a risk factor (aOR 4.43, CI 1.42–17.02, *p*‐value 0.016), and age (aOR 0.94, CI 0.91–0.98, *p*‐value 0.001) and being hospitalised in the dementology unit (aOR 0.40, CI 0.19–0.80, *p*‐value 0.01) were found to be protective factors for BZRA prescription at admission. Baseline characteristics and determinant factors for BZD alone are presented in Tables 4 and 5, respectively. Baseline characteristics and determinant factors for z‐drugs alone are presented in Tables 6 and 7, respectively.

**TABLE 2:** Baseline characteristics of patients with at least one prescription of BZRA and patients without prescription of BZRA (*N* = 386)

	Without prescription	With prescription	Total
Sex, *N* (%)			
Female	138 (64.5)	76 (35.5)	214 (100)
Male	119 (69.2)	53 (30.8)	172 (100)
Total	257 (66.6)	129 (33.4)	386 (100)
Primary diagnosis, *N* (%)			
F0	125 (76.7)	38 (23.3)	163 (100)
F1	4 (22.2)	14 (77.8)	18 (100)
F2	23 (57.5)	17 (42.5)	40 (100)
F3	69 (62.2)	42 (37.8)	111 (100)
F4	19 (61.3)	12 (38.7)	31 (100)
F5	0 (NA)	0 (NA)	0 (NA)
F6	15 (71.4)	6 (28.6)	21 (100)
F7	1 (100)	0 (0.0)	1 (100)
F8	1 (100)	0 (0.0)	1 (100)
F9	0 (NA)	0 (NA)	0 (NA)
Total	257 (66.6)	129 (33.4)	386 (100)
Dementia MCI, *N* (%)			
Dementia	156 (74.3)	54 (25.7)	210 (100)
MCI	19 (61.3)	12 (38.7)	31 (100)
None	82 (56.6)	63 (43.4)	145 (100)
Total	257 (66.6)	129 (33.4)	386 (100)
Unit of hospitalisation, *N* (%)			
PSY	70 (54.7)	58 (45.3)	128 (100)
GEN1	32 (72.7)	12 (27.3)	44 (100)
GEN2	75 (64.1)	42 (35.9)	117 (100)
DEM	80 (82.5)	17 (17.5)	97 (100)
Total	257 (66.6)	129 (33.4)	386 (100)
Age (years)			
*n*	257	129	386
Mean	79.7	75.1	78.2
SD	8.2	7.1	8.1

DEM, dementology; GEN1, GEN2, two general psychogeriatric units; MCI, mild cognitive impairment; PSY for unit of hospitalisation.

**TABLE 3:** Results of logistic regression.

	aOR	2.5%	97.5%	*p*‐Value
Sex (Ref = Female)				
Male	0.751	0.463	1.211	0.242
Age (years)				
Age (years)	0.944	0.912	0.976	0.001
Primary diagnosis (Ref = F3)				
F0	0.975	0.442	2.186	0.950
F1	4.433	1.416	17.022	0.016
F2	0.963	0.435	2.099	0.925
F4	1.139	0.475	2.668	0.767
F6	1.056	0.319	3.213	0.925
Dementia/MCI (Ref = None)				
Dementia	0.891	0.415	1.897	0.764
MCI	1.053	0.431	2.514	0.908
Unit of hospitalisation (Ref = PSY)				
GEN1	0.590	0.258	1.292	0.196
GEN2	0.862	0.464	1.603	0.639
DEM	0.396	0.192	0.794	0.010

aOR are presented along with their 95% CI and *p*‐value. For categorical variables, levels are compared with a reference, which is female for sex, F3 for the primary diagnosis, no impairment for cognitive status and PSY for unit of hospitalisation. DEM, dementology; MCI, mild cognitive impairment.

**TABLE 4:** Baseline characteristics of patients with at least one prescription of BZD and patients without prescription of BZD (*N* = 386)

	Without prescription	With prescription	Total
Sex, *N* (%)			
Female	147 (68.7)	67 (31.3)	214 (100)
Male	127 (73.8)	45 (26.2)	172 (100)
Total	274 (71.0)	112 (29.0)	386 (100)
Primary diagnosis, *N* (%)			
F0	130 (79.8)	33 (20.2)	163 (100)
F1	4 (22.2)	14 (77.8)	18 (100)
F2	24 (60.0)	16 (40.0)	40 (100)
F3	79 (71.2)	32 (28.8)	111 (100)
F4	20 (64.5)	11 (35.5)	31 (100)
F5	0 (NA)	0 (NA)	0 (NA)
F6	15 (71.4)	6 (28.6)	21 (100)
F7	1 (100)	0 (0.0)	1 (100)
F8	1 (100)	0 (0.0)	1 (100)
F9	0 (NA)	0 (NA)	0 (NA)
Total	274 (71.0)	112 (29.0)	386 (100)
Dementia/MCI, *N* (%)			
Dementia	162 (77.1)	48 (22.9)	210 (100)
MCI	20 (64.5)	11 (35.5)	31 (100)
None	92 (63.4)	53 (36.6)	145 (100)
Total	274 (71.0)	112 (29.0)	386 (100)
Unit of hospitalisation, *N* (%)			
PSY	78 (60.9)	50 (39.1)	128 (100)
GEN1	33 (75.0)	11 (25.0)	44 (100)
GEN2	78 (66.7)	39 (33.3)	117 (100)
DEM	85 (87.6)	12 (12.4)	97 (100)
Total	274 (71.0)	112 (29.0)	386 (100)
Age (years)			
*n*	274	112	386
Mean	79.5	75	78.2
SD	8.1	7.3	8.1

DEM, dementology; MCI, mild cognitive impairment.

**TABLE 5:** Results of logistic regression.

	aOR	2.5%	97.5%	*p*‐Value
Sex (Ref = Female)				
Male	0.698	0.419	1.151	0.161
Age (years)				
Age (years)	0.942	0.908	0.976	0.001
Primary diagnosis (Ref = F3)				
F0	1.290	0.562	3.032	0.552
F1	6.754	2.148	26.041	0.002
F2	1.376	0.609	3.068	0.437
F4	1.608	0.649	3.876	0.294
F6	1.602	0.473	5.021	0.429
Dementia/MCI (Ref = None)				
Dementia	0.981	0.442	2.158	0.963
MCI	1.357	0.536	3.344	0.511
Unit of hospitalisation (Ref = PSY)				
GEN1	0.677	0.287	1.515	0.354
GEN2	0.943	0.498	1.787	0.857
DEM	0.311	0.137	0.669	0.004

aOR are presented along with their 95% CI and *p*‐value. For categorical variables, levels are compared with a reference, which is female for sex, F3 for the primary diagnosis, no impairment for cognitive status and PSY for unit of hospitalisation. aOR, adjusted odds ratio; DEM, dementology; MCI, mild cognitive impairment.

**TABLE 6:** Baseline characteristics of patients with at least one prescription of z‐drug and patients without prescription of z‐drug (*N* = 386)

	Without prescription	With prescription	Total
Sex, *N* (%)			
Female	198 (92.5)	16 (7.5)	214 (100)
Male	155 (90.1)	17 (9.9)	172 (100)
Total	353 (91.5)	33 (8.5)	386 (100)
Primary diagnosis, *N* (%)			
F0	156 (95.7)	7 (4.3)	163 (100)
F1	13 (72.2)	5 (27.8)	18 (100)
F2	37 (92.5)	3 (7.5)	40 (100)
F3	97 (87.4)	14 (12.6)	111 (100)
F4	27 (87.1)	4 (12.9)	31 (100)
F5	0 (NA)	0 (NA)	0 (NA)
F6	21 (100)	0 (0.0)	21 (100)
F7	1 (100)	0 (0.0)	1 (100)
F8	1 (100)	0 (0.0)	1 (100)
F9	0 (NA)	0 (NA)	0 (NA)
Total	353 (91.5)	33 (8.5)	386 (100)
Dementia/MCI, *N* (%)			
Dementia	199 (94.8)	11 (5.2)	210 (100)
MCI	28 (90.3)	3 (9.7)	31 (100)
None	126 (86.9)	19 (13.1)	145 (100)
Total	353 (91.5)	33 (8.5)	386 (100)
Unit of hospitalisation, *N* (%)			
PSY	113 (88.3)	15 (11.7)	128 (100)
GEN1	40 (90.9)	4 (9.1)	44 (100)
GEN2	109 (93.2)	8 (6.8)	117 (100)
DEM	91 (93.8)	6 (6.2)	97 (100)
Total	353 (91.5)	33 (8.5)	386 (100)
Age (years)			
*n*	353	33	386
Mean	78.5	74.9	78.2
SD	8.3	6	8.1

DEM, dementology; MCI, mild cognitive impairment.

**TABLE 7:** Results of logistic regression.

	aOR	2.5%	97.5%	*p*‐Value
Sex (Ref = Female)				
Male	1.399	0.649	3.037	0.390
Age (years)				
Age (years)	0.981	0.926	1.037	0.506
Primary diagnosis (Ref = F3)				
F0	0.343	0.089	1.354	0.120
F1	2.396	0.650	8.031	0.166
F2	0.528	0.114	1.804	0.349
F4	0.957	0.251	3.000	0.944
Dementia/MCI (Ref = None)				
Dementia	0.948	0.263	3.073	0.932
MCI	0.754	0.161	2.622	0.683
Unit of hospitalisation (Ref = PSY)				
GEN1	0.876	0.228	2.743	0.831
GEN2	0.750	0.260	2.043	0.580
DEM	0.912	0.285	2.667	0.870

aOR are presented along with their 95% CI and *p*‐value. For categorical variables, levels are compared with a reference, which is female for sex, F3 for the primary diagnosis, no impairment for cognitive status and PSY for unit of hospitalisation. aOR, adjusted odds ratio; DEM, dementology; MCI, mild cognitive impairment.

### 3.5 Associations between change in BZRA prescription and hospital LOS

**FIGURE 3:**
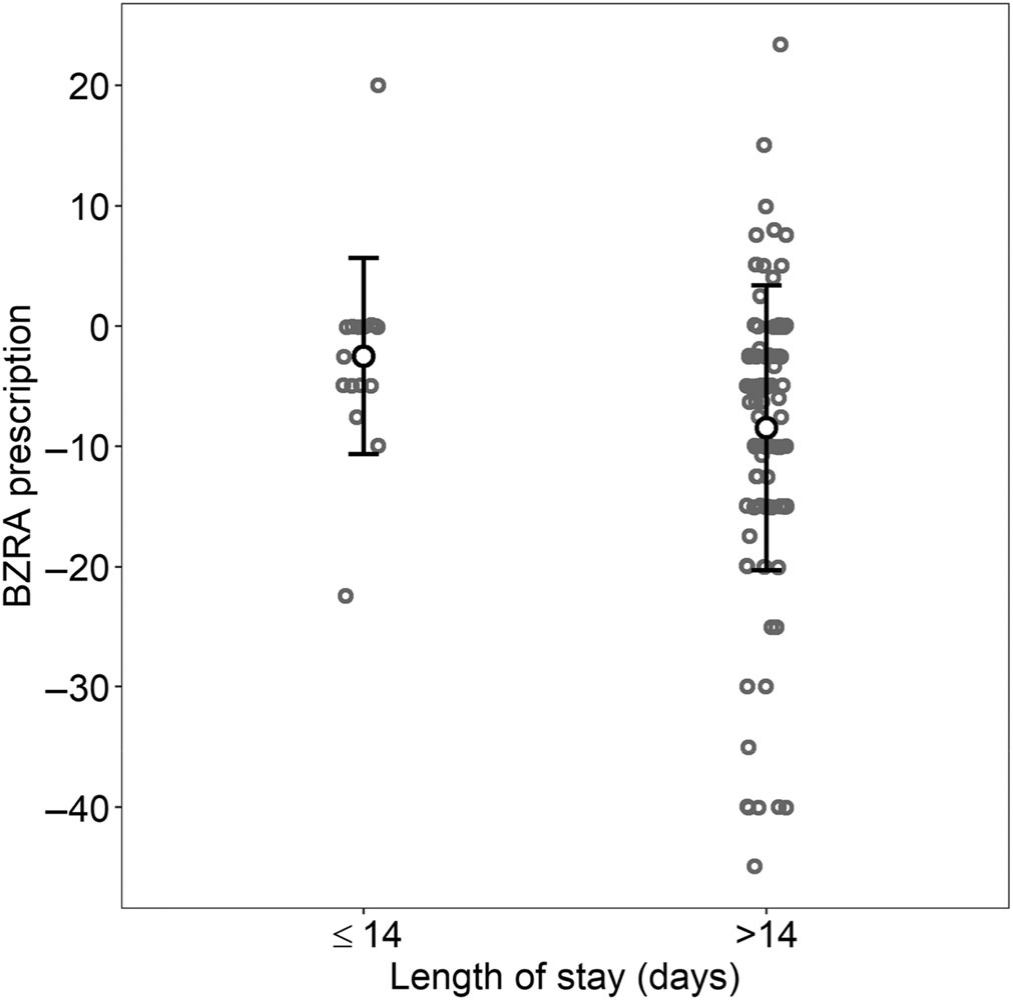
Changes in benzodiazepine‐receptor agonists (BZRA) prescription (in standardised dose of mg of diazepam) depending on the length of hospital stay (≤ 14 days versus 14 days). Mean and standard deviation (SD) are represented.

The BZRA prescription in standardised dose was compared between hospital stays of ≤ 14 days and hospital stays of > 14 days using linear regression and adjusting for the unit of hospitalisation. Hospital stays of > 14 days had a statistically significantly higher reduction of BZRA in standardised dose than hospital stays of < 14 days (difference of −4.76, CI −8.91, −0.61 mg of diazepam, *p*‐value 0.025), as shown in Figure 3.

### 3.6 Factors predicting change in BZRA prescription at discharge

**FIGURE 4:**
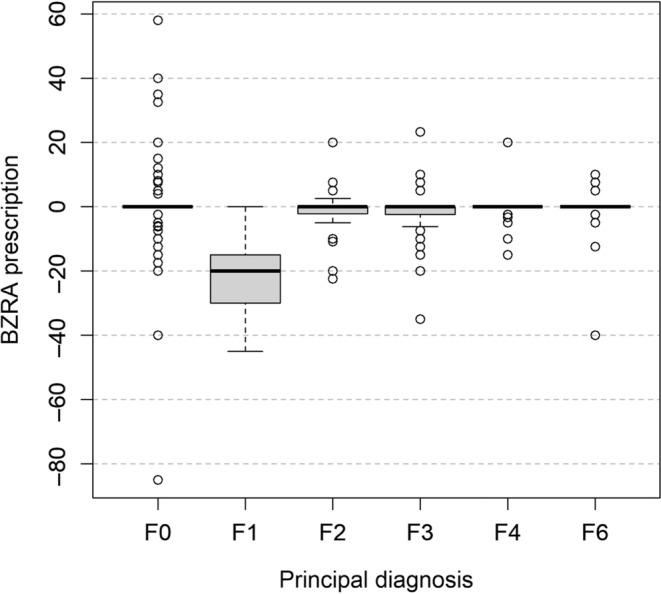
Change in benzodiazepine‐receptor agonists (BZRA) prescription (in standardised dose of mg of diazepam) between admission and discharge by main diagnosis. No patients had a main diagnosis of F5 or F9, only one patient had a main diagnosis of F7, and only one patient had a main diagnosis of F8. Patients with a main diagnosis of F7 and F8 were thus removed from the analysis and were not considered for the descriptive statistics (the total number of patients is then *n* = 384).

**FIGURE 5:**
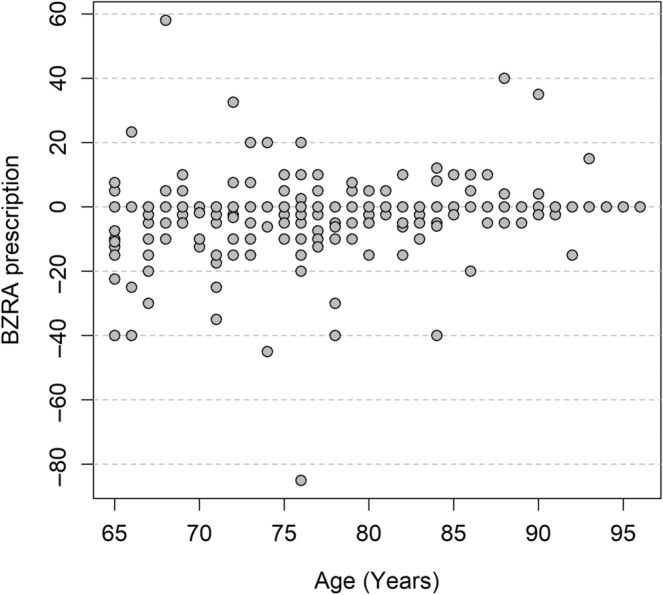
Change in benzodiazepine‐receptor agonists (BZRA) prescription (in standardised dose) between admission and discharge by age.

When taking into account standardised dose of BZRA, primary diagnosis of F1 (mental and behavioural disorders due to psychoactive substance use) was found to be a factor predicting reduction at discharge as compared with admission (mean of −18.63, CI −23.32, −13.95 mg of diazepam, *p*‐value < 0.0001), as illustrated in Figure 4. Age was not statistically significant as a factor predicting increase (mean of +0.10, CI −0.03, 0.23, *p*‐value 0.13), as shown in Figure 5.

When taking into account change in BZRA prescription during hospital stay in terms of decrease of dose versus no change or increase, primary diagnosis of F1 was again found to be a factor predicting reduction at discharge as compared with admission (aOR 7.96, CI 2.53, 30.62, *p*‐value 0.001), and age was found to be a factor predicting an increase (aOR 0.95, CI 0.92, 0.99, *p*‐value 0.012).

## 4 DISCUSSION

### 4.1 Main findings

This study was an observational retrospective study evaluating the prescription of BZRA at admission and at discharge in a population of patients hospitalised in four psychogeriatric units in a Swiss university hospital. The main findings of the study were that one out of three patients (33.4%) in our sample received at least one type of BZRA at admission against one out of five (22.5%) at discharge, and that the total standardised dose of BZRA was significantly reduced at discharge compared with admission (relative reduction of 78%). Secondarily, a main diagnosis of F1 (mental and behavioural disorders due to use of psychoactive substances) was associated with a higher risk of BZRA prescription at admission (risk factor), and age and being hospitalised in the dementology unit were associated with a lower risk of BZRA prescription at admission (protective factor). We also found a main diagnosis of F1 to be a factor predicting reduction of BZRA at discharge compared with admission, and age to be a factor predicting increase. Finally, regarding hospital LOS, we found that hospital stays > 14 days were associated with a higher reduction of BZRA in standardised dose than hospital stays of ≤ 14 days.

To the best of our knowledge, there are no studies assessing the prevalence of BZRA prescription in hospitalised psychogeriatric patients, or in hospitalised psychiatric patients. Our study showed that 33.4% of patients hospitalised in one of the psychogeriatric units of our university hospital received BZRA at admission against 22.5% at discharge. Although that does not reflect what patients received right before their hospital stay or right after (our study defined admission as the second day of hospital stay and discharge as the penultimate day), we observed a much higher prevalence of BZRA prescription than that found in the general population. Abolhassani et al. (2019), working with a general population cohort in Lausanne, the capital city of the region of our study, found a prevalence of BZRA use in the general population of 8.4%, which increased up to 13.6% in the population aged 65 years or older. Interestingly, they found a higher risk of BZRA use in people presenting with anxiety (relative risk [RR] 5.61, CI 3.61–8.71) and depression (RR 3.75, CI 2.47–5.69), with a prevalence of BZRA use of 27% and 17.8%, respectively, closer to the prevalence of BZRA prescription in our study.

Concerning determinant factors for BZRA prescription, Abolhassani et al. (2019) found female gender to be associated with a higher use of BZRA (RR of 1.80, CI 1.14–2.85, compared with male), which we did not find in our study. A different study by Luta et al. (2020) evaluating patterns of benzodiazepine prescription among older adults enrolled in a specific insurance company in Switzerland found an increase of prevalence of benzodiazepine prescription with age, which differs from our findings of age being a protective factor for benzodiazepine (and BZRA in general) prescription at admission.

Regarding the finding of hospitalisation in the dementology unit being a protective factor, but not the diagnosis of dementia itself, we could hypothesise that health professionals working in this unit were more aware of the risk of adverse effects of BZRA in this especially fragile population and tended to prescribe them less frequently. Nevertheless, a study by Rijksen et al. (2021) studying the prevalence of benzodiazepines and z‐drugs in nursing home residents with dementia in the Netherlands found that 39.2% of residents were prescribed BZRA, with a large proportion of these prescriptions not following the guidelines with regard to indication, suggesting that working with this specific population alone could not explain for a better adhesion to guidelines and recommendations.

Regarding our finding of a main diagnosis of F1 being a risk factor for BZRA prescription at admission, we did not find other studies in hospital settings for comparison. Nonetheless, because benzodiazepines are the best evidence‐based treatment for alcohol and BZRA withdrawal (Brett & Murnion, 2015; Kattimani & Bharadwaj, 2013), and because many patients suffering from mental and behavioural disorders due to use of psychoactive substances consume primarily or concomitantly benzodiazepines and z‐drugs (Roehrs & Roth, 2012), it was not a surprising finding. The same principle applies to F1 being a factor predicting BZRA reduction at discharge compared with admission, as the treatment for alcohol or BZRA withdrawal consists in slowly tapering down (and eventually eliminating) benzodiazepines (Brett & Murnion, 2015; Kattimani & Bharadwaj, 2013). That could also explain the important reduction of oxazepam at discharge when compared with admission seen in Figure 2 as oxazepam is one of the most frequently used drugs to treat alcohol withdrawal in inpatients, and therefore it is introduced at the beginning of the hospitalisation and slowly tapered down until, if possible, stopped (de Millas et al., 2010; Holbrook et al., 1999).

As far as the type of molecule is concerned, apart from oxazepam the study found a high prevalence of prescription of lorazepam both at admission and at discharge. Lorazepam is in fact one of the most widely used benzodiazepines in psychiatric settings for its fast onset and high potency, which usually makes it the benzodiazepine of choice to treat acute anxiety, acute insomnia and agitation (Hui, 2018). Regarding the other prescribed molecules, we observe a switch at discharge compared with admission for longer‐acting benzodiazepines with lower abuse potential (Grandjean et al. 2021; Schmitz, 2016).

Regarding the higher reduction of BZRA in standardised dose in hospitalisations longer than 14 days compared with shorter ones, we did not find other studies for comparison, although we could hypothesise that longer hospitalisations allow for more important BZRA reductions, especially for those patients hospitalised for BZRA or alcohol withdrawal (Holbrook et al., 1999). Also, acute symptoms like anxiety tend to alleviate after some days of hospitalisation (Kathol & Wenzel, 1992), suggesting that the medication can be reduced and adapted to the changing needs of patients.

As far as we know, there are no studies evaluating the evolution of BZRA prescription throughout a hospitalisation (neither general nor psychiatric hospitalisation), except for those evaluating a specific intervention to reduce BZRA prescription during hospitalisations. Nonetheless, new BZRA prescriptions after hospitalisation are frequent in older adults, and they may result in chronic use of these substances (Bell et al., 2007; Heinemann et al., 2019). In fact, a study by Schumacher et al. (2017) evaluating the prescription of sedative drugs (BZRA and other substances) during hospital stays in a Swiss general hospital found an absolute increase of 10% of these prescriptions (58% of which were BZRA), which differs from our findings, showing a decrease in the number of prescriptions. Nevertheless, both populations are not comparable, as the population in Shumacher's study included patients of all ages and hospitalised for medical causes, and our study included only patients aged 65 years or more hospitalised for psychiatric causes.

### 4.2 Strengths and limitations

To the best of our knowledge, this is the first study focusing on the prescription of benzodiazepines and z‐drugs in this specific population, patients hospitalised in psychogeriatric units. With the fast aging of the Swiss population (and most of Occidental populations) and the growing prevalence of dementia, more studies in this field are needed.

One of the main strengths of our study was the fact that, being a retrospective observational study with information extracted from the electronic medical information system, we had no missing data, which made our statistical calculations stronger. Our study provided a large sample of 386 patients and, because we did not find similar studies to calculate the necessary sample for the study to have significance, taking a whole natural year as reference seemed relevant. More importantly, our study included real‐life patients, aged 65 years or older, with a mean age of 78.2 ± 8.1 years. These patients are often excluded from clinical trials because of high comorbidity or polypharmacy (Trotter, 2002), but at the same time they represent the majority of real‐life patients. Therefore, in order to generate evidence‐based medicine, it seems relevant to include them in research studies.

On the other hand, this is an observational study, which has its limitations. The above‐mentioned risk and protective factors and the factors predicting change concern this specific sample, but no generalisations can be made. Nonetheless, these kind of studies are important for the generation of hypotheses, which can later on be tested in higher‐quality studies.

Another important limitation was the fact that the reason for BZRA prescription was not specified and, although BZRA are often prescribed for symptoms of insomnia, both benzodiazepines and z‐drugs have other indications, both psychiatric (e.g. anxiety, agitation, catatonia) and neurological (e.g. epilepsy). On the other hand, only BZRA were included in the study, although other molecules such as sedative antipsychotics, sedative antidepressants, antihistaminic drugs and others are often prescribed off‐label for insomnia symptoms, and we could not evaluate for an eventual switching effect of one sedative‐hypnotic drug for another one.

Despite these limitations, we could infer from our data that BZRA prescription in this population was not in line with current insomnia guidelines recommending CBT‐I as a first‐line treatment for insomnia disorder (Riemann et al., 2023). Nonetheless, we could argue that these guidelines are relevant for insomnia disorder only, and guidelines for the treatment of acute insomnia symptoms do not exist. In any case, a recent study has found a lack of implementation of CBT‐I in psychiatric facilities (Schneider et al., 2023). Nevertheless, promising strategies are being tested to implement an adapted version of CBT‐I in psychiatric hospitals for adults (Schneider et al., 2020). To the best of our knowledge, specific behavioural interventions for psychogeriatric inpatients or inpatients with cognitive decline or severe somatic comorbidities have not been tested.

Finally, an important limitation of our study was the fact that BZRA status was based on the status on the second and penultimate day, so it did not reflect what patients received right before the hospitalisation and right after. Unfortunately, the information regarding the patients' medication at home was not systematically documented, neither was the patients' medication at discharge, and this option seemed preferable to reflect the reality of what happened during the hospitalisation.

## 5 CONCLUSION

Our study showed a high prevalence of BZRA prescription at admission, which decreased at discharge both in absolute and in relative terms, with longer hospitalisations being associated with a higher reduction of BZRA prescription. Having a main diagnosis of F1 (mental and behavioural disorders due to psychoactive substance use) was found to be a risk factor for BZRA prescription at admission, while age and being hospitalised in the dementology unit were found to be protective factors.

Prescription of BZRA in our population of hospitalised psychiatric patients was high, and probably not in line with current guidelines recommending CBT‐I as a first‐line treatment for insomnia disorder. Non‐pharmacological strategies, including behavioural treatment, for patients, their carers and the health teams, should be further developed in this specific population.

## AUTHOR CONTRIBUTIONS

**Maria Dalmau i Ribas:** Conceptualization; methodology; validation; writing – original draft; writing – review and editing. **Julien Sauser:** Methodology; software; data curation; validation; formal analysis; writing – original draft; writing – review and editing. **Estelle Gillès de Pélichy:** Conceptualization; methodology; supervision; writing – review and editing; validation. **Montserrat Méndez Rubio:** Writing – review and editing; validation. **Jean‐Pierre Schuster:** Writing – review and editing; validation. **Armin Von Gunten:** Validation; writing – review and editing. **José Haba‐Rubio:** Conceptualization; methodology; validation; supervision; writing – review and editing; funding acquisition; project administration.

## Data Availability

The data that support the findings of this study are available on request from the corresponding author. The data are not publicly available due to privacy or ethical restrictions.
